# Using Motivated Cue Integration Theory to Understand a Moment-by-Moment Transformative Change: A New Look at the Focusing Technique

**DOI:** 10.3389/fnhum.2018.00307

**Published:** 2018-08-14

**Authors:** Idit Shalev

**Affiliations:** Laboratory for Embodiment and Self-Regulation, Department of Psychology, Ariel University, Ariel, Israel

**Keywords:** focusing, self-regulation, cue integration, embodied-cognition, stress, automaticity, sensation, awareness

The practice of mind-body techniques in therapy or self-development promotes the transformation of experience from stress to resilience by using techniques that involve awareness of bodily sensation. However, little is known about the underlying mechanisms that effect the transition from stress to resilience. Much of the theory and research indicate the efficacy of top-down emotion regulation strategies of stressful experiences (Salovey and Mayer, 1990; Gross, 2014; Schäfer et al., 2017), and less is known about moment-by-moment awareness of sensation techniques. Based on the perspective of motivated cue integration (Shalev, 2015), the effect of perceptual cues on the creation of stress and resilience will be presented. The importance of awareness of sensation as a mechanism that enables resilience will be discussed using the principles that underlie the practice of focusing as laid out by Gendlin (1986, 1996, 2012).

## Embodied cognition and automatic self-regulation

Individuals' psychological experience is influenced by their momentary interpretations of perceptual cues (Loersch and Payne, 2011). Theories of embodied cognition suggest that bodily sensations and physical environmental cues are stored as analogous psychological concepts in memory, and their activation by such cues automatically spreads from their physical experiences to their metaphorically related psychological representations and vice versa (Wilson, 2002; Niedenthal et al., 2005; Meier et al., 2012). In other words, higher level processing is grounded in the organism's lower level sensory and motor experiences (Wilson, 2002; Barsalou, 2010; Meier et al., 2012; Winkielman et al., 2015), indicating that psychological experience is associated with sensual characteristics. For example, research has demonstrated that automatic activation of either the physical or metaphoric representation of a homeostatic cue (e.g., thirst, temperature) similarly influenced self-regulation without conscious awareness (Shalev, 2014, 2016; Halali et al., 2017). Despite these and other findings about how nonconscious cues influence self-regulation (Shalev and Bargh, 2011), little is known about the effects, if any, that an awareness of sensation may have on one's ability to overcome stress through resilience. To address this gap in the literature, I start by explaining how motivated cue integration affects psychological experience (Shalev, 2015), after which I will introduce the unique properties of motivated cue integration that render it highly applicable in the context of stress. Finally, I will demonstrate the moment-by-moment change from stress to resilience using the focusing technique (Gendlin, 2012).

## Theory of motivated cue integration: the creation of experience

The theory of motivated cue integration (Shalev, 2015) suggests that the individual's momentary system of goals (Kruglanski et al., 2002) modulates perceptual cue-integration. Goals are defined as cognitive representations of desired end states that affect evaluations, emotions and behaviors (Fishbach and Ferguson, 2007). As such, one's active goals qualitatively affect her selective attention to multisensory information that, in turn, influences the creation of meaning and action generation (Shalev, 2015) in both bottom up and top down processes (Zaki, 2013). For example, exposure to a signal of threat (e.g., a gun at school) will activate an avoidance goal that, on the one hand, will increase the likelihood of sensing the relevant embodied and perceptual cues (e.g., increased heart rate, sweating, flashbacks of past shooting). On the other hand, the probability that the perceiver will infer that a shooting will occur may be proportional to the strengths of the associations between the contextual cue of the gun and the unique interpretation of the observer.

Indeed, the theory of motivated cue integration proposes that internal and external cue integrations are influenced by the perceiver's automatic motivational constraints (Shalev, 2015). In the context of a gun at school, for example, a mental representation of a violent attack would be created in the perceiver's mind only if the gun carries motivational relevance for them, that is, to prevent undesired results (e.g., assassination) or to establish a picture of what is real (e.g., shooting) (Eitam and Higgins, 2010). Another motivational constraint is the individual's unique structure of goals and means that, in turn, is organized around an associative structure unique to that individual (Wertheimer, 1923, p. 625). For example, in the repeated coupling of sensory signals (Rescorla, 1985), the strength of the association between a specific bodily sensation and a psychological concept may influence judgment and behavior (e.g., physical and social warmth or coldness) (Bargh and Shalev, 2012), and the proximity of bodily cues and the temporal contiguity of multisensory cues may similarly influence interpretation (Helson, 1933). Additionally, a third motivational constraint comprises the individual's mental resources (Kahneman, 1973), which influence the effort invested in cue integration and subsequent action generation.

## The unique properties of stress experiences

Following the general principles of motivated cue integration theory, the specific bodily experience of stress is determined by unique automatic behavior-organizing properties that help explain the need for awareness of sensation as a transformative change mechanism. For example, there is evidence that the internalized structure of fear is characterized by cognitive representations of the stimulus typical of the fear situation, the individual's responses in it, aspects of its meaning for the individual, and the individual's autonomic arousal and mood state (Foa and Kozak, 1986), all of which characteristics manifest as an automatic pattern. Likewise, research indicates that stress is associated with a low tolerance for ambiguity and a high need for cognitive closure that are expressed through a desire for predictability, a preference for order and structure, and close-mindedness (Webster and Kruglanski, 1994; Roets et al., 2015). The bodily responses to stress are fight, flight or freeze, increased heart rate or enhanced blood flow to skeletal muscles (Bracha, 2004). Moreover, emotional distress, low emotional awareness and poor emotion regulation might aggravate the physical reaction to stress (Bonn-Miller et al., 2011). Research suggests that undifferentiated emotions tend to propagate somatic stress symptoms through implicit emotional processing (Lane and Schwartz, 1987). Deficits in one's awareness and expression of emotions are assumed to constitute one of the disconnection syndromes (Lane, 2008), which describe a pathological uncoupling between the higher-level, neocortical emotional processes and subcortical emotion-generating processes. This critical missing connection contributes to dysfunctions in the autonomic nervous system (ANS) and hypothalamic-pituitary-adrenal (HPA) axis (MacLean, 1949), thus leading to homeostatic dysregulation and eventually to disease (Kanbara and Fukunaga, 2016). In what follows, I will describe the technique of focusing that is used to increase one's awareness of sensation, examine the mechanism that underlies this technique and explore its association with motivated cue integration theory.

## The focusing technique

“Focusing” is a process and a teachable skill developed by Gendlin (1996) in which the client learns how to attend to vague, physical impressions until they become well-defined and meaningful. The client is encouraged to sense the yet unformed feelings and associative meanings of experiences to obtain inner resonance and to verify ideas and feelings, such that new and fresh insights emerge (Gendlin, 1986). Through a taught, step-by step process, focusing teaches one how to orient attention inwards and effectively rewire bodily awareness to enable one to exploit the self-knowledge inherent to body and mind.

Key to focusing is the felt sense, an unclear, pre-verbal sense of “something” meaningful—the inner knowledge or awareness of which has yet to be consciously thought or verbalized—as that “something” is experienced in the body. This bodily felt, initially nondescript “something” may be an awareness of a situation or of something that is “coming”—perhaps an idea or insight. Crucial to the concept, as defined by Gendlin (1996), is that it is unclear and vague, and any attempt to express it verbally usually results in its trivialization.

The focusing procedure begins with the focuser being guided to monitor or sense her bodily signals, during which she is instructed to allow semantic cues (e.g., memories, mental images, emotions, etc.) to emerge slowly from this sensing. From among what emerges, the focuser is requested to select one personal concern on which to concentrate and to allow herself to explore its low-clarity representation in the present mind-body experience. To accurately identify its meaning, the focuser then tries to assign words, mental images or phrases that may express the present experience. These words or images can be tested against the bodily felt sense, which will not resonate with a word or phrase that does not adequately describe it. Once the focuser has accurately identified this felt sense in words, new words or images emerge that give new insight into the situation. Eventually, there will be a sense of felt movement—a “felt shift”—as the person gains some clarity about the felt sense and begins to move beyond the “stuck” place. In addition to fresh insights, the focuser may also obtain some indications of steps that can be taken to address her current concern. Next, the focuser is asked to remain in touch with the experience for several minutes, even if it only confers on her a slight release of tension. Because whatever emerges from the focusing activity, it only represents the clarification of a single, undefined experience, there will be others (Gendlin, 2012). An alternative approach to focusing uses the quality of “radical acceptance” as an invitation by the client to simultaneously relate to different perhaps contradicting aspects of the experience (Cornell, 2008), and in so doing, she can experience a broader, more holistic cue integration process.

## Effect of the focusing technique on motivated cue integration: a moment-by-moment embodied self-regulation

Following the example of a stressful experience, an examination of the focusing technique from the perspective of the motivated cue integration theory suggests that change of the “top down” goal (e.g., avoidance) to self-exploration goal will influence perceptual cue integration. Whereas a stressful experience is characterized by a high need for cognitive closure based on the known preference of conservative predictable judgment (Webster and Kruglanski, 1994; Roets et al., 2015), the self-exploration goal encourages attention to changes in bodily sensations, enabling the emergence of fresh, new details with which to reconstruct the stressful psychological experience. A simultaneous bottom-up explorative process is activated by selectively attending to distinct internal cues that increase the individual's awareness of her unique motivational constraints (e.g., strength of association, type of mental images, metaphors). One's use of selective attention while remaining in the moment facilitates the generation of new associations that compete with existing conditioning patterns and may subsequently provide valuable new insight into the current situation. Thus, the process of devoting one's attention to peripheral marginal signals and then engaging in their valuation begets the opportunity for cue re-integration. Once the “felt shift” has been experienced, the practice of remaining with the current experience provides the opportunity to memorize the process as an integral part of long-term memory.

## Focusing and other “awareness of sensation” techniques

The practice of focusing could be associated with other techniques for augmenting awareness of sensation. For example, attention to newly available information is a mechanism common to both focusing and mindfulness. Moreover, philosophical traditions such as Buddhism entail the cultivation of a moment-to-moment, nonjudgmental awareness of one's present experience (Kabat-Zinn, 2003). An additional mechanism shared by the techniques of focusing and mindfulness is an attitude of acceptance and interest in moment-by-moment cues (Shapiro et al., 2006). Whereas mindfulness in the Eastern tradition (Kabat-Zinn, 2003) exploits meditation that emphasizes suspending the myriad ways of interpreting experience and attending instead to the experience itself, the practice of focusing (Gendlin, 2012) and the Western notion of mindfulness (Langer, 1989) encourage interest in and engagement with the creation of meaning. The creation of new meaning and its assignment to old patterns may lead not only to marked reductions in stress; it may also involve substantive alterations in the architecture of one's motivated cue-integration. Acknowledging the potential strengths of this technique, it was recently adopted by practitioners of accelerated psychodynamic therapy (AEDP, Fosha, 2000), a therapeutic approach that draws heavily on the concept and process of focusing. Altogether, by encouraging the client to develop the capacity to explore vague, bodily, and emotional states, the awareness of sensation technique facilitates a creative process of motivated cue re-integration that fosters increased resilience and better self-regulation (Brown and Ryan, 2003), value clarification and cognitive flexibility (Shapiro et al., 2006).

## Author contributions

The author confirms being the sole contributor of this work and approved it for publication.

### Conflict of interest statement

The author declares that the research was conducted in the absence of any commercial or financial relationships that could be construed as a potential conflict of interest.

## References

[B1] Bargh J. A. Shalev I. (2012). The substitutability of physical and social warmth in daily life. Emo 12, 154–162. 10.1037/a0023527 21604871 PMC3406601

[B2] Barsalou L. W. (2010). Grounded cognition: past, present, and future. Top. Cog. Sci. 2, 716–724. 10.1111/j.1756-8765.2010.01115.x 25164052

[B3] Bonn-Miller M. O. Vujanovic A. A. Boden M. T. Gross J. J. (2011). Posttraumatic stress, difficulties in emotion regulation, and coping-oriented marijuana use. Cog. Behav. Ther, 40, 34–44. 10.1080/16506073.2010.525253 21337213

[B4] Bracha H. S. (2004). Freeze, flight, fight, fright, faint: adaptationist perspectives on the acute stress response spectrum. CNS Spect. 9, 679–685. 10.1017/S1092852900001954 15337864

[B5] Brown K. W. Ryan R. M. (2003). The benefits of being present: mindfulness and its role in psychological well-being. J. Per. Soc. Psych. 84:822. 10.1037/0022-3514.84.4.822 12703651

[B6] Cornell A. W. (2008). The Focusing Teacher's Manual: Module One, Working With Focusing Clients One-to-One. Berkeley, CA: Calluna Press.

[B7] Eitam B. Higgins E. T. (2010). Motivation in mental accessibility: relevance of a representation (ROAR) as a new framework. Soc. Pers. Psych. Comm. 4, 951–967. 10.1111/j.1751-9004.2010.00309.x 21116462 PMC2992320

[B8] Fishbach A. Ferguson M. J. (2007). The goal construct in social psychology, in Social Psychology: Handbook of Basic Principles, eds Kruglanski A. W. Higgins E. T. (New York, NY: Guilford), 490–515.

[B9] Foa E. B. Kozak M. J. (1986). Emotional processing of fear: exposure to corrective information. Psych. Bull. 99:20. 10.1037/0033-2909.99.1.20 2871574

[B10] Fosha D. (2000). The Transforming Power of Affect: A Model for Accelerated Change. Basic Books.

[B11] Gendlin E. T. (1986). What comes after traditional psychotherapy research? Amer. Psy. 41:131. 10.1037/0003-066X.41.2.131 3963609

[B12] Gendlin E. T. (1996). Focusing-Oriented Psychotherapy: A Manual of the Experiential Method. New York, NY: Guilford Press.

[B13] Gendlin E. T. (2012). Focusing-Oriented Psychotherapy: A Manual of the Experiential Method. Guilford Press.

[B14] Gross J. J. (2014). Emotion regulation: conceptual and empirical foundations. J. Cognit. Neurosci. 2014, 3–20.

[B15] Halali E. Meiran N. Shalev I. (2017). Keep it cool: temperature priming effect on cognitive control. Psy. Res. 81, 343–354. 10.1007/s00426-016-0753-6 26910519

[B16] Helson H. (1933). The fundamental propositions of Gestalt psychology. Psych. Rev. 40, 13–32. 10.1037/h0074375

[B17] Kabat-Zinn J. (2003). Mindfulness-based interventions in context: past, present, and future. Clin. Psych. Sci. Prac. 10, 144–156. 10.1093/clipsy.bpg016

[B18] Kahneman D. (1973). Attention and Effort. Englewood Cliffs, NJ: Prentice-Hall.

[B19] Kanbara K. Fukunaga M. (2016). Links among emotional awareness, somatic awareness and autonomic homeostatic processing. Bio. Psych. Soc. Med. 10:16. 10.1186/s13030-016-0059-3 27175214 PMC4863353

[B20] Kruglanski A. W. Shah J. Y. Fishbach A. Friedman R. Chun W. Y. Sleeth-Keppler D. (2002). A theory of goal systems. Adv. Exp. Soc. Psych. 34, 331–378. 10.1016/S0065-2601(02)80008-9

[B21] Lane R. D. (2008). Neural substrates of implicit and explicit emotional processes: a unifying framework for psychosomatic medicine. Psychos. Med. 70, 214–231. 10.1097/PSY.0b013e3181647e44 18256335

[B22] Lane R. D. Schwartz G. E. (1987). Levels of emotional awareness: a cognitive-developmental theory and its application to psychopathology. Am. J. Psych. 144, 133–143. 3812780 10.1176/ajp.144.2.133

[B23] Langer E. J. (1989). Mindfulness. Addison-Wesley/Addison Wesley Longman.

[B24] Loersch C. Payne B. K. (2011). The situated inference model an integrative account of the effects of primes on perception, behavior, and motivation. Pers. Psych. Sci. 6, 234–252. 10.1177/1745691611406921 26168515

[B25] MacLean P. D. (1949). Psychosomatic disease and the visceral brain; recent developments bearing on the Papez theory of emotion. Psy. Med. 11, 338–353. 10.1097/00006842-194911000-00003 15410445

[B26] Meier B. P. Schnall S. Schwarz N. Bargh J. A. (2012). Embodiment in social psychology. Top. Cog. Sci. 4, 705–716. 10.1111/j.1756-8765.2012.01212.x 22777820

[B27] Niedenthal P. M. Barsalou L. W. Winkielman P. Krauth-Gruber S. Ric F. (2005). Embodiment in attitudes, social perception, and emotion. Per. Soc. Psy. Rev. 9, 184–211. 10.1207/s15327957pspr0903_1 16083360

[B28] Rescorla R. A. (1985). Pavlovian conditioning analogues to Gestalt perceptual principles, in Affect, Conditioning, and Cognition: Essays on the Determinants of Behavior, 113–130.

[B29] Roets A. Kruglanski A. W. Kossowska M. Pierro A. Hong Y. Y. (2015). The motivated gatekeeper of our minds: New directions in need for closure theory and research. Adv. Exp. Soc. Psychol. 52, 221–283. 10.1016/bs.aesp.2015.01.001

[B30] Salovey P. Mayer J. D. (1990). Emotional intelligence. Imag. Cog. Per. 9, 185–211. 10.2190/DUGG-P24E-52WK-6CDG

[B31] Schäfer J. Ö. Naumann E. Holmes E. A. Tuschen-Caffier B. Samson A. C. (2017). Emotion regulation strategies in depressive and anxiety symptoms in youth: a meta-analytic review. J. Youth. Adol. 46, 261–276. 10.1007/s10964-016-0585-0 27734198

[B32] Shalev I. (2014). Implicit energy loss: embodied dryness cues influence vitality and depletion. J. Consum. Psy. 24, 260–270. 10.1016/j.jcps.2013.09.011

[B33] Shalev I. (2015). The architecture of embodied cue integration: insight from the “motivation as cognition” perspective. Front. Psychol. 6:658. 10.3389/fpsyg.2015.00658 26052294 PMC4440347

[B34] Shalev I. (2016). Pictorial and mental arid landscape images reduce the motivation to change negative habits. J. Env. Psy. 45, 30–39. 10.1016/j.jenvp.2015.11.005

[B35] Shalev I. Bargh J. A. (2011). Use of priming-based interventions to facilitate psychological health: commentary on Kazdin and Blase (2011). Pers. Psy. Sci. 6, 488–492. 10.1177/1745691611416993 26168201

[B36] Shapiro S. L. Carlson L. E. Astin J. A. Freedman B. (2006). Mechanisms of mindfulness. J. Clin. Psych. 62, 373–386. 10.1002/jclp.20237 16385481

[B37] Webster D. M. Kruglanski A. W. (1994). Individual differences in need for cognitive closure. J. Per. Soc. Psy. 67:1049. 10.1037/0022-3514.67.6.1049 7815301

[B38] Wertheimer M. (1923). Untersuchungen zur Lehre von der Gestalt II, in Psycologische Forschung, Vol. 4, 301–350. 10.1007/BF00410640

[B39] Wilson M. (2002). Six views of embodied cognition. Psy. Bull. Rev. 9, 625–636. 10.3758/BF03196322 12613670

[B40] Winkielman P. Niedenthal P. Wielgosz J. Eelen J. Kavanagh L. C. (2015). Embodiment of cognition and emotion, in APA Handbook of Personality and Social Psychology, Attitudes and Social Cognition, eds Mikulincer M. Shaver P. R. Borgida E. Bargh J. A. (Washington, DC: American Psychological Association), 151–175.

[B41] Zaki J. (2013). Cue integration a common framework for social cognition and physical perception. Pers. Psychol. Sci. 8, 296–312. 10.1177/1745691613475454 26172972

